# Applications of Functional Magnetic Resonance Imaging in Determining the Pathophysiological Mechanisms and Rehabilitation of Spatial Neglect

**DOI:** 10.3389/fneur.2020.548568

**Published:** 2020-11-12

**Authors:** Yuqian Zhang, Yan Hua, Yulong Bai

**Affiliations:** ^1^Department of Rehabilitation Medicine, Huashan Hospital, Fudan University, Shanghai, China; ^2^Department of Rehabilitation Medicine, Huashan Hospital North, Fudan University, Shanghai, China

**Keywords:** functional magnetic resonance imaging, dorsal attention network, ventral attention network, interhemispheric rivalry, pathophysiological mechanisms, spatial neglect, rehabilitation

## Abstract

Functional magnetic resonance imaging (fMRI) is a neuroimaging tool which has been applied extensively to explore the pathophysiological mechanisms of neurological disorders. Spatial neglect is considered to be the failure to attend or respond to stimuli on the side of the space or body opposite a cerebral lesion. In this review, we summarize and analyze fMRI studies focused specifically on spatial neglect. Evidence from fMRI studies have highlighted the role of dorsal and ventral attention networks in the pathophysiological mechanisms of spatial neglect, and also support the concept of interhemispheric rivalry as an explanatory model. fMRI studies have shown that several rehabilitation methods can induce activity changes in brain regions implicated in the control of spatial attention. Future investigations with large study cohorts and appropriate subgroup analyses should be conducted to confirm the possibility that fMRI might offer an objective standard for predicting spatial neglect and tracking the response of brain activity to clinical treatment, as well as provide biomarkers to guide rehabilitation for patients with SN.

## Introduction

“Spatial neglect” (SN) is a contralesional spatial bias (i.e., the failure to attend or respond to stimuli on the side of the space or body opposite the lesion) (1). It is correlated with impaired vigilance/arousal that results in delayed responses and non-spatial deficits in attentional capacity (2).

SN can occur subsequent to neurodegenerative disease (3, 4), cancer (5), trauma (6), but it occurs most commonly subsequent to stroke (7, 8). Research on patients with stroke has shown that SN occurs in 43% of right brain-lesioned patients and 20% of left brain-lesioned patients at baseline (8). At 3 months, SN continues to be present in 17% of right brain-lesioned patients and 5% of left brain-lesioned patients (8). SN negatively affects motor and cognitive function, activities of daily living (ADL), and duration of hospital stay (9–12).

SN is a heterogeneous syndrome and has several subtypes, such as perceptual vs. intentional (13), personal space vs. extra-personal space (14), or egocentric vs. allocentric representation (15). A battery of neuropsychological tests is administered to assess SN symptoms in the clinic, including conventional pencil-and-paper tests [e.g., line bisection, cancellation, copying or drawing figures, reading/writing; (16)], and ecological evaluations [e.g., Catherine Bergego Scale, Behavioral Inattention Test, Subjective Neglect Questionnaire, Baking Tray Task, wheelchair obstacle course, ADL-based SN battery; (17, 18)]. These tests can assess various classes of SN symptoms, and different patients may show deficits in different subsets of tests.

## Functional Magnetic Resonance Imaging (fMRI)

fMRI is a neuroimaging tool that employs MRI to image regional, time-varying changes in neural activity (19). The blood-oxygen-level-dependent (BOLD) signal is detected in fMRI. The BOLD signal represents an indirect measure of neuronal activity through neurovascular coupling. The latter is a cascade of physiological processes linking local neuronal activity to orchestrated changes in local blood flow and blood oxygenation (20). Researchers can document specific signal changes from the entire brain in a relatively short time during a specific task or at rest, which contributes to the popularity of fMRI in neuroscience research. The increasing popularity of fMRI also derives from its non-invasive nature and excellent spatial resolution.

Task-based fMRI and resting-state fMRI are the two main types of fMRI. Task-based fMRI is acquired while the individual is instructed to perform a particular task, such as a motor task, social cognition, working memory, incentive processing, emotion processing, language processing, attention, or object location (21). Task-based fMRI is highly dependent upon the applied task, which is a minor drawback. Resting-state fMRI measures spontaneous, low-frequency fluctuations (<0.1 Hz) in the BOLD signal without a task or stimulus (22). The individual should stay still and avoid cognitive, language or motor tasks with eyes either closed or open or staring at a fixed point while data are acquired (23, 24). Widely separated (though functionally related) brain regions showing temporally correlated fluctuations constitute a brain functional network. Functional networks identified through resting-state fMRI can be identified similarly through task-based fMRI (25, 26). Also, measurements of the temporal correlation of BOLD signals between different brain regions in the resting state, called “resting-state functional connectivity,” can be used to map topography between different brain networks (27, 28).

Two main limitations of fMRI impede its wide application in the clinic. First, the BOLD signal can be affected by several factors: movements or tasks during scans; temperature and technical noise of the MR system; hormonal rhythms, blood pressure and heart rate of individual; diet; time of day (23, 29). This variability in the BOLD signal affects the reliability of task-based fMRI and resting-state fMRI. Second, a lack of standardized acquisition and analytical methods also hinder its use for diagnostic purposes (30).

## Pathophysiological Mechanisms Underlying SN

**Figure 1:**
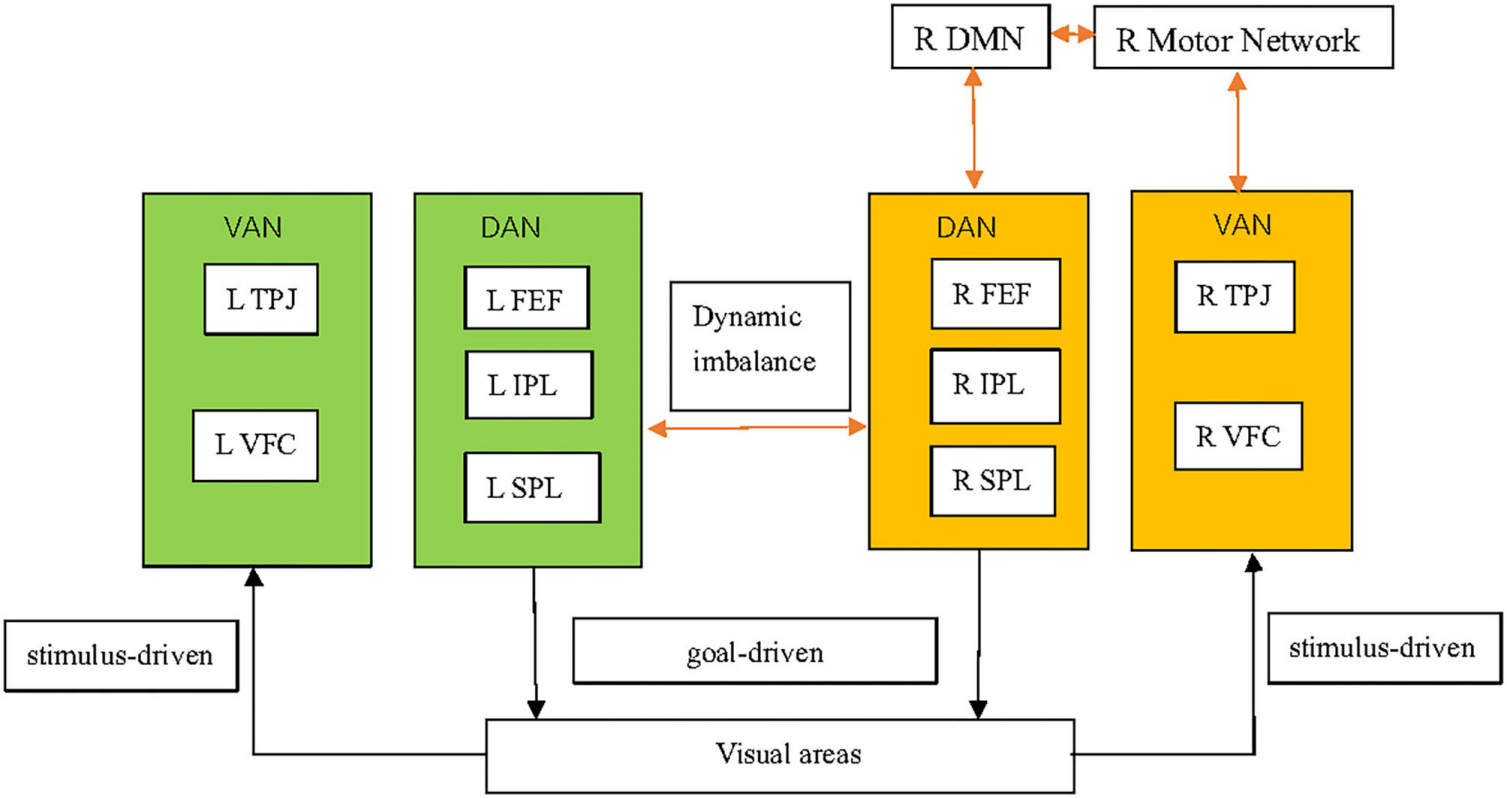
Pathophysiological mechanisms underlying spatial neglect. The DAN, which mainly comprises the SPL, IPS, and FEFs, is a goal-driven, “top–down” network and supports top–down endogenous attention. The VAN, which mainly comprises the TPJ and VFC, is a stimulus-driven or “bottom–up” network and is implicated in detecting exogenous task-relevant stimuli. Structural damage in VAN regions can induce DAN dysfunction. A virtual lesion on the DAN induced by transcranial magnetic stimulation can exert DAN dysfunction. SN might involve not only dysfunction of the DAN and VAN, but also other brain functional networks, including the DMN and motor networks. SN patients have decreased inter-network connectivity between the VAN and motor network. SN patients also have decreased inter-network connectivity between the DAN/motor network and the DMN in the right hemisphere. Functional imbalance of brain activity in left (hyperactive) and right (hypoactive) brain regions involved in the control of spatial attention is observed in SN patients, which supports the concept of the interhemispheric-rivalry mechanism as an explanatory model. The shading in orange and green indicate, respectively, relative decreases and increases in functional activity. The orange arrows indicate decreased connectivity between networks. DAN, dorsal attention network; VAN, ventral attention network; SPL, superior parietal lobe; IPS, intraparietal sulcus; FEFs, frontal eye fields; TPJ, temporal–parietal junction; VFC, ventral frontal cortex; DMN, default mode network.

SN is dependent not only upon localized damage to specific brain structures but also the function of brain regions that are far from the local lesion (31). Brain networks are referred to sets of brain regions that contribute to the performance of a particular set of functions, or set of related tasks (32). Increasingly, fMRI studies have explored the effects of stroke on brain networks, and linked abnormalities in those networks on the behavioral deficits in SN [(31, 33, 34); Figure 1]. Evidence from fMRI has also demonstrated the validity of “interhemispheric rivalry mechanisms” as an explanatory model of SN [(31, 35); Figure 1]. The concept of interhemispheric rivalry proposed originally by Kinsbourne suggests that each hemisphere contains a processor of spatial attention for the contralateral visual field, and reciprocal transcallosal inhibition has been postulated to underlie the balance of attention toward the left vs. right visual fields (36).

### Dysfunction of Attention Networks

Attention networks comprise the dorsal attention network (DAN) and ventral attention network (VAN). Dysfunction of attention networks has a critical role in SN (37–40). The DAN is composed mainly of the superior parietal lobe (SPL), intraparietal sulcus (IPS), and frontal eye fields (FEFs). The DAN is a goal-driven, “top–down” network and supports top–down endogenous attention based on prior knowledge, expectations, and current goals (38, 41, 42). The VAN mainly comprises the temporal–parietal junction (TPJ) and ventral frontal cortex (VFC). The VAN is a stimulus-driven or “bottom–up” network and is implicated in detecting task-relevant sensory events, particularly if they are unexpected (43–45). The TPJ includes the posterior sector of the superior temporal sulcus (STS) and superior temporal gyrus (STG), as well as the ventral part of the supramarginal gyrus (SMG). The VFC includes the inferior frontal gyrus (IFG) and the middle frontal gyrus (MFG) in the anterior insula (AI), and frontal operculum. If attention is reoriented to a new target, a reorienting signal from the VAN interrupts the ongoing task in the DAN, which shifts attention toward the novel source of information (46). Furthermore, the DAN contributes to the suppression of exogenous task-irrelevant information (41). Thus, except perhaps when individuals are not carrying out an ongoing task, the VAN cannot be activated by exogenous task-irrelevant stimuli, but environmental task-relevant stimuli. The posterior parietal cortex (PPC) (including the IPS and TPJ) interacts with the visual cortex for the selection of relevant targets (46).

SN is observed commonly in stroke patients with lesions restricted to the right VAN (31, 47). However, it can also occur if lesions are restricted to the component of the DAN, such as the IPS (48). There is a complex interaction between these two attention networks during target detection under sustained spatial attention. Lesions in parts of the VAN have been shown to evoke profound activation changes in parts of the structurally intact DAN measured by resting-state functional connectivity MRI which, in turn, correlates with SN severity in stroke patients (39, 49). The fractional amplitude of low-frequency fluctuations of the BOLD signal in resting-state fMRI in the structurally intact right SPL (as part of the DAN) is strongly correlated with the SN-related functional impairment and pathological attention bias in SN patients with structural damage to the VAN (39). Studies using task-based fMRI have found that an anatomically intact DAN (especially the IPS and SPL) of damaged hemispheres shows weak or no task-related activity during the Posner Cuing Task in SN patients with VAN lesions (31). In short, physiological dysfunction of the DAN induced by structural damage to the VAN is observed not only at rest, but also during task performance by fMRI. SN-like symptoms can be evoked temporally by transcranial magnetic stimulation (TMS) to model the virtual lesion on the dorsal right parietal cortex in healthy individuals (50). One task-based fMRI study demonstrated that TMS, as a causal perturbation approach on the IPS (a critical component of the DAN), could exert profound directional causal influences on the TPJ (a critical component of the VAN) during target detection under sustained spatial attention (51).

Increasing numbers of fMRI studies have reported that SN might involve not only attention networks but also other brain functional networks (34, 40, 52). One study on resting-state functional connectivity found that SN patients had decreased interhemispheric connectivity in the VAN and decreased inter-network connectivity between the VAN and the motor network (primary motor cortices) (52). Moreover, Baldassarre et al. found that SN in first-ever stroke patients was associated with correlated multi-network patterns of abnormal functional connectivity by resting-state fMRI (34). Specifically, the DAN and sensory–motor networks showed a loss of intra-hemispheric anti-correlation with the default mode network (DMN) in the right hemisphere (34). Also, improvement of attention deficits was correlated with a restoration of the normal anti-correlation between dorsal attention/motor regions and the DMN, as assessed by resting-state functional connectivity, particularly in the damaged hemisphere (40). The DMN encompasses a group of discrete, bilateral, symmetrical and functionally connected brain regions, in the medial and lateral parietal, medial prefrontal, as well as medial and lateral temporal cortices (53). It exhibits higher levels of activity during relaxed states than during performance of externally oriented cognitive tasks (54). Another study based on resting-state fMRI focused specifically on attention deficits and motor deficits. The authors demonstrated that behavioral attention deficits were associated significantly more with decreased interhemispheric functional connectivity of the DAN than with motor networks, whereas motor deficits were associated significantly more with decreased interhemispheric functional connectivity of motor networks than with the DAN (33).

### Model of Interhemispheric Rivalry

Supporting evidence for the model of interhemispheric rivalry stems from clinical observation of a patient who suffered from sequential strokes in both hemispheres with severe SN after a first right-sided parietal infarct and abrupt disappearance of SN after a second left-sided frontal infarct (55). Emerging evidence from fMRI supports the validity of interhemispheric-rivalry mechanisms as an explanatory model of SN (31, 34, 35, 40, 49).

Functional imbalance of task-evoked brain activity in the left (hyperactive) and right (hypoactive) dorsal parietal cortex has been observed in patients with SN after right frontal damage, even though these areas were structurally intact (31). The hyperactivity of the unaffected hemisphere might result from a loss of inter-hemispheric inhibition, which may be reflected by interhemispheric functional connectivity using resting-state functional connectivity MRI. Furthermore, an imbalance in the interhemispheric coherence of regions involved in the control of spatial attention (as measured by resting-state functional connectivity MRI) correlates with deficits in spatial attention (35). One study based on functional connectivity fMRI acquired fMRI data while study participants undertook an event-related attention task, but with the deterministic task-evoked effects removed, to conduct functional connectivity analyses. The authors found that interhemispheric connectivity in the posterior IPS was disrupted acutely but recovered at the chronic stage. This finding was in accordance with the observation that the right posterior IPS was less recruited than the left posterior IPS at the acute stage, and that they returned to balanced activation at the chronic stage in patients with SN (49). Also, improvement of attention deficits was closely related to increases in inter-hemispheric functional connectivity between regions of the dorsal attention, motor, visual, and auditory networks as assessed by resting-state functional connectivity MRI (40). In conclusion, SN involves the relative hyperactivity of the unaffected hemisphere due to release from reciprocal inhibition by the affected hemisphere. Also, improvement of attention deficits is correlated with rebalance in brain regions implicated in the control of spatial attention between the unaffected and affected hemispheres, which suggests the validity of the interhemispheric-rivalry model.

TMS can deactivate the brain cortex temporally to model the virtual lesion and to induce SN-like behaviors in healthy people. The validity of interhemispheric-rivalry mechanisms has also been supported by studies on task-based fMRI and resting-state fMRI using TMS to induce SN-like behaviors in healthy individuals (56, 57). Petitet et al. used TMS to transiently inhibit activity in the right caudal part of the angular gyrus at the junction with the IPS, and assessed the changes of functional brain activity by task-based fMRI during a bilateral target-detection task (56). They demonstrated that the direction of TMS-induced attentional bias and changes in brain activity (i.e., the leftward shift in parietal activity and rightward shift in attentional bias) was consistent, which suggested that the balance of functional brain activity between the left and right parietal cortex determined the spatial allocation of attention (56). Wang and colleagues, using repetitive TMS and resting-state functional connectivity fMRI, indicated that a TMS-induced unbalanced interaction between the interhemispheric top–down network of posterior SPL and V1 correlated with lateralization of visuospatial attention in healthy individuals (57). The interhemispheric-rivalry model has also been supported by evidence showing that behavioral deficits of SN patients were improved when TMS over the left PPC or left frontal cortex was employed to normalize the over-excitability of the contralesional hemisphere for interhemispheric rebalance (58, 59).

## Rehabilitation Methods

SN affects the rehabilitation of other stroke-related symptoms negatively, and is associated with reduced functional independence in ADL (60). Development of efficacious treatment strategies for SN provides an important opportunity to improve the functional outcome of stroke (60–62). Consensus on the most efficacious therapy for SN is lacking. However, several promising interventions have been proposed to improve SN symptoms: prismatic adaptation (PA), non-invasive brain stimulation (NIBS), motor imagery (MI), optokinetic stimulation (OKS) and virtual reality (VR) (61–76). Besides, there is a low level of evidence in favor of mirror therapy, neck-muscle vibration, family involvement, motor activation and spatial cueing for SN (77). These methods can be classified into three main types: top–down, bottom–up, and modulation of intracerebral inhibition processes [i.e., NIBS; (77)]. Top–down methods are based on a voluntary effort of the patient following a therapist's instructions, such as MI (77). Bottom–up methods are based on the patient's sensory environment or visuomotor adaptation, such as PA and OKS (61, 71). NIBS–TMS and transcranial direct current stimulation (tDCS), which were developed on a model of interhemispheric competition, can also ameliorate the behavioral deficits of SN by reducing the activity of the unaffected hemisphere or by increasing the activity of the affected hemisphere (78, 79). fMRI has been used as an additional assessment of existing therapy strategies to evaluate changes in the neural activity of the brain cortex. In this review, we focus on changes in fMRI signals induced by the promising rehabilitation methods mentioned above.

### PA

PA can be used to alleviate SN (64, 71, 80–87). PA is dependent upon the mismatch between the perceived position of a target seen through prismatic goggles and its real position relative to the body (68). PA includes two adaptive processes: recalibration (which contributes to early error correction) and spatial realignment (which contributes to after-effect development). In a typical protocol for PA, individuals wear prismatic goggles with a rightward deviation of the visual image and perform tasks to reach visual targets. At first, participants will miss the real target, pointing erroneously in the direction of prismatic displacement. With repeated pointing movements, participants can adapt gradually to the prismatic displacement and correct their errors to reach the real target. After removal of the prisms, participants exhibit pointing errors in the direction opposite to the prismatic shift, which denotes the adaptation “after-effect” (88).

fMRI studies have investigated the effect of PA on brain activation in healthy people to determine visuomotor plasticity, which can indirectly explain neural substrates underlying the therapeutic benefits of PA. One study using resting-state fMRI explored transient changes of resting-state functional connectivity in the right DAN induced by a session of pointing tasks with a prism in healthy people. The authors found that a rightward prism modulated resting-state functional connectivity between the right IPS and FEFs belonging to the right DAN and functional connectivity between the right anterior cingulate cortex and FEFs (89). Furthermore, Wilf et al. discovered that PA-induced functional modulation was not limited within attention networks, but was characterized instead by enhancement of the decoupling between the DMN and DAN/VAN. Their findings were based on comparison of patterns of resting-state functional connectivity before and after a 3-min pointing task with a rightward-shifting prism in healthy individuals (90). The cerebellum is also involved in visuomotor adaptation (91–94).

Some fMRI studies investigated changes in brain activity induced by PA to directly explain neural substrates underlying the therapeutic effect of PA on patients with SN. One task-based fMRI study examined the effect of PA by comparing brain activity during three tasks (bisection, search, and memory) before and after a single PA session (95). The authors found increased activation in the bilateral PPC, mid-frontal cortex, and occipital cortex during bisection and visual-searching tasks accompanied by significant behavioral improvement (95). Another study on task-based fMRI using a detection task reported that the PA-induced alleviation of SN predominately involved the left superior temporal region (64). The fMRI studies mentioned above suggest that the beneficial effect of PA on SN is derived from modulation of cortical regions implicated in spatial cognition in damaged and undamaged hemispheres. Furthermore, the effect of PA on SN is dependent upon the site of brain damage. Compared with SN patients with parietal lesions, PA can induce a higher level of improvement in SN patients with frontal lesions, accompanied by enhanced activity of posterior parietal and mid-frontal areas bilaterally, as measured by task-based fMRI during bisection and search tasks (96). Differences in brain plasticity induced by PA were documented among the fMRI studies mentioned above. More fMRI studies in SN patients with different lesion sites and subtypes of SN are needed to explain the underlying neural mechanisms of PA.

### OKS

OKS is a promising therapeutic method that can induce enduring and functionally relevant positive effects in patients with right-hemisphere stroke and SN (97). OKS requires patients to perform smooth-pursuit eye movements following visual stimuli that move coherently from the ipsilesional to the contralesional side on a screen. OKS can lead to an exogenously triggered directing of spatial attention to the neglected side.

Several fMRI studies in healthy individuals have provided evidence that OKS can induce almost symmetrical activations in multiple brain regions, including frontoparietal regions (FEFs, IPS) as well as the primary and associated visual cortices, insula, basal ganglia, cerebellum, and brainstem in both hemispheres independent of the stimulus direction (98–100).

Some fMRI studies investigating the neural mechanisms of OKS in SN patients have shown inconsistent results. One study based on task-based fMRI found that OKS led to increased neural activity bilaterally in the middle frontal gyrus and precuneus. In addition, OKS activated the cingulate gyrus, middle temporal gyrus, angular gyrus and occipital cortex in the left hemisphere as measured by task-based fMRI during a spatial-attention task, accompanied by amelioration of SN symptoms in SN patients suffering from chronic right-hemisphere lesions (97). A compensatory recruitment of left-hemisphere areas induced by OKS contributes to SN amelioration in stroke patients with chronic right-hemisphere lesions. In patients with an acute right-hemisphere stroke, leftward OKS led to mostly bilateral activations in the visual cortex (V1–V4), IPS, FEFs, supplementary eye fields and thalamus, as measured by task-based fMRI during OKS, which was negatively correlated with behavioral impairment (101). The differences in neural activity induced by OKS among the two fMRI studies mentioned above might be because the study participants were in different stages of stroke.

### MI

MI involves the mental execution of an action in the absence of movement. MI can be used as a complement to physical training for stroke patients (102, 103).

MI can recruit brain networks consisting of premotor regions [e.g., IFG and supplementary motor area (SMA)], parietal regions (e.g., SMG, IPL, SPL), and subcortical regions (e.g., putamen and cerebellum) in healthy people (104, 105). However, few scholars have investigated the changes in brain activity induced by MI in SN patients. One pilot study examined neuronal activation by task-based fMRI in patients with chronic SN with right-hemispheric stroke during MI whereby patients had to imagine touching each of their four fingers with the tip of the thumb (106). The authors found that the left primary somatosensory, premotor cortices and SMA were activated during MI of the unaffected hand. However, MI of the affected hand was related to activations in the left premotor cortex, left AI, left dorsolateral prefrontal cortex, medial SMA, right rolandic operculum and right SPL (106). The authors also revealed that SN severity was positively related to brain activation in the SMA during MI of the affected hand (106). Although the results should be treated with caution due to absence of a matched control group and small sample size, they suggested that MI can activate functionally relevant brain areas in SN patients.

### TMS

Repetitive transcranial magnetic stimulation (rTMS) is a method of NIBS. rTMS may be useful for exploring the SN pathophysiology and ameliorating its symptoms (107). Low-frequency rTMS (≤1 Hz) lowers cortical excitability, whereas high-frequency rTMS (≥10 Hz) increases cortical excitability, likely by modulating neurotransmitters such as gamma-aminobutyric acid and dopamine. Theta burst stimulation (TBS), a variant of rTMS, involves application of short trains of stimuli at high frequency repeated at intervals of 200 ms (108). TBS can be subclassified as intermittent theta-burst stimulation (iTBS) and continuous TBS (cTBS) based on the pattern of stimulation (109). iTBS influences motor-evoked potentials to produce long-term potentiation, whereas cTBS induces prolonged depression of brain activity.

The mechanism underlying SN might involve the relative hyperactivity of the unaffected hemisphere due to release from reciprocal inhibition by the affected hemisphere (35, 49). Thus, inhibitory low-frequency rTMS or cTBS over the unaffected PPC could improve SN (78, 79). One case report revealed that cTBS applied over the left PPC improved SN symptoms in one patient with traumatic brain injury (110). That observation was associated with decreased excitability of the PPC–M1 connections in the left hemisphere and bilateral increased functional connectivity in the frontoparietal network shown by resting-state fMRI (110). Thus, the authors considered that cTBS could have partially reduced the interhemispheric imbalance due to a decrease in the hyper-excitability of the left PPC-M1 connections and increased connectivity in frontal-parietal networks.

Several studies have explored the effect of high-frequency rTMS or iTBS on SN. A double-blind, sham-controlled study compared the therapeutic effect of low- and high-frequency rTMS applied over the PPC. The authors found that high-frequency rTMS over the lesioned PPC improved SN significantly more than low-frequency rTMS over the non-lesioned PPC in patients with acute stroke (111). Cao and colleagues applied iTBS to the left dorsal lateral prefrontal cortex in patients with right-hemisphere stroke and SN (112). They found that increasing the activity of the left dorsal lateral prefrontal cortex through iTBS could ameliorate SN symptoms (112). They also found that iTBS at a resting motor threshold of 80% induced a large-scale reduction in the extent of resting-state functional connectivity, mostly in right attention networks, and more significant amelioration of behavioral performance compared with iTBS at a resting motor threshold of 40% (112).

### VR

VR is a computer-based, multisensory, stimulating, and interactive environment that occurs in real-time whereby the individual is engaged in activities that appear similar to real-world objects and events (113, 114). VR combines top–down and bottom–up treatments for SN. RehAtt™, a novel multisensory VR device, can combine scanning training in a three-dimensional game with intense multisensory stimulation (70). Fordell et al. found that 5-week RehAtt training improved spatial attention and ADL in older patients with chronic SN (70).

A pilot study used task-based fMRI to evaluate the change in brain activity during the Posner Cuing Task before and after RehAtt intervention in patients with chronic SN (115). They demonstrated that 5-week RehAtt training improved performance in patients with chronic SN and increased their brain activity during cue-induced focus of attention in the prefrontal cortex (e.g., dorsolateral prefrontal cortex and anterior cingulate cortex), middle and superior temporal gyrus, but showed no training effects during target presentations (115). Another pilot study revealed that 5-week iRehAtt intervention improved SN symptoms, and increased inter-hemispheric resting-state functional connectivity in the DAN between the right FEF and left IPS in patients with chronic SN as measured by resting-state fMRI (116). The studies mentioned above suggest that VR is a promising approach to post-stroke management of SN due to changes in relative brain activities. However, a more extensive prospective controlled study is needed to explain the different results among studies, and to obtain a potential marker that would allow a priori identification of patients as responders or non-responders for VR training.

## Conclusions

fMRI opens the way for greater understanding of the pathophysiological mechanisms underlying SN and potentially improves our ability to evaluate the effect of rehabilitation methods. fMRI studies have demonstrated that SN might result from abnormal changes in attention networks and other brain functional networks, including the DMN and motor network. Several promising interventions (PA, NIBS, MI, OKS, and VR) could modulate the cortical regions implicated in spatial cognition measured by fMRI, which might contribute to a beneficial effect to the clinical presentation of SN.

However, differences between fMRI studies have been documented, and caution needs to be taken in utilizing their conclusions due to three main reasons. First, SN is a heterogeneous syndrome and can be fractionated into several subtypes. However, fMRI studies have employed mostly small study cohorts and included patients were barely divided into different subgroups of SN. Second, task-based fMRI is highly dependent upon the applied task during scans, but task-based fMRI studies have used different tasks while fMRI is acquired. Third, acquisition and analytical methods of fMRI influence its results. Thus, fMRI cannot yet offer an objective standard for diagnosing or predicting SN in the clinic. fMRI can be used to track the response of SN to various treatments in clinical trials, but it cannot provide a biomarker to determine which treatment is most appropriate for a specific subtype of SN in the clinic.

Prospective controlled studies or randomized controlled trials of large sample size, appropriate subgroup analyses, as well as standard acquisition and analytical methods of fMRI should be conducted. In this way, fMRI might prognosticate the risk of SN, track its response of brain activity to treatment, and provide biomarkers to guide rehabilitation for patients with SN.

## Author Contributions

YZ wrote the first draft. YB and YH provided critical revisions. All authors contributed to the article and approved the submitted version.

## Conflict of Interest

The authors declare that the research was conducted in the absence of any commercial or financial relationships that could be construed as a potential conflict of interest.

## Abbreviations

ADL: activities of daily living
AI: anterior insula
AG: angular gyrus
BOLD: blood-oxygen-level-dependent
DAN: dorsal attention network
DMN: default mode network
FEFs: frontal eye fields
fMRI: functional magnetic resonance imaging
IFG: inferior frontal gyrus
IPL: inferior parietal lobule
IPS: intraparietal sulcus
MFG: middle frontal gyrus
MI: motor imagery
NIBS: non-invasive brain stimulation
OKS: optokinetic stimulation
PA: prismatic adaptation
PPC: posterior parietal cortex
SMG: supramarginal gyrus
SMA: supplementary motor area
SN: spatial neglect
SPL: superior parietal lobe
STS: superior temporal sulcus
STG: superior temporal gyrus
TMS: transcranial magnetic stimulation
TPJ: temporal–parietal junction
VAN: ventral attention network
VFC: ventral frontal cortex
VR: virtual reality.
